# Supplementary material for: Characterizing the effective reproduction number during the COVID-19 pandemic: Insights from Qatar’s experience
Source: J Glob Health. 2022 Feb 5;12:05004. doi: 10.7189/jogh.12.05004 (PMC8819337; doi:10.7189/jogh.12.05004)
Supplement: Online Supplementary Document [file jogh-12-05004-s001.pdf]

**Online Supplementary Document**

# Characterizing the effective reproduction number during the COVID-19 epidemic: Insights from Qatar's experience

**Figure S1.** A conceptual diagram illustrating the basic structure of the deterministic mathematical model developed to describe SARS-CoV-2 transmission dynamics in the population of Qatar. The detailed structure of this model and its description are found in [1,2]. In this figure, solid lines denote progression or forward movement from one population compartment to the next, while dashed lines denote backward movement from the present population compartment to the previous one.

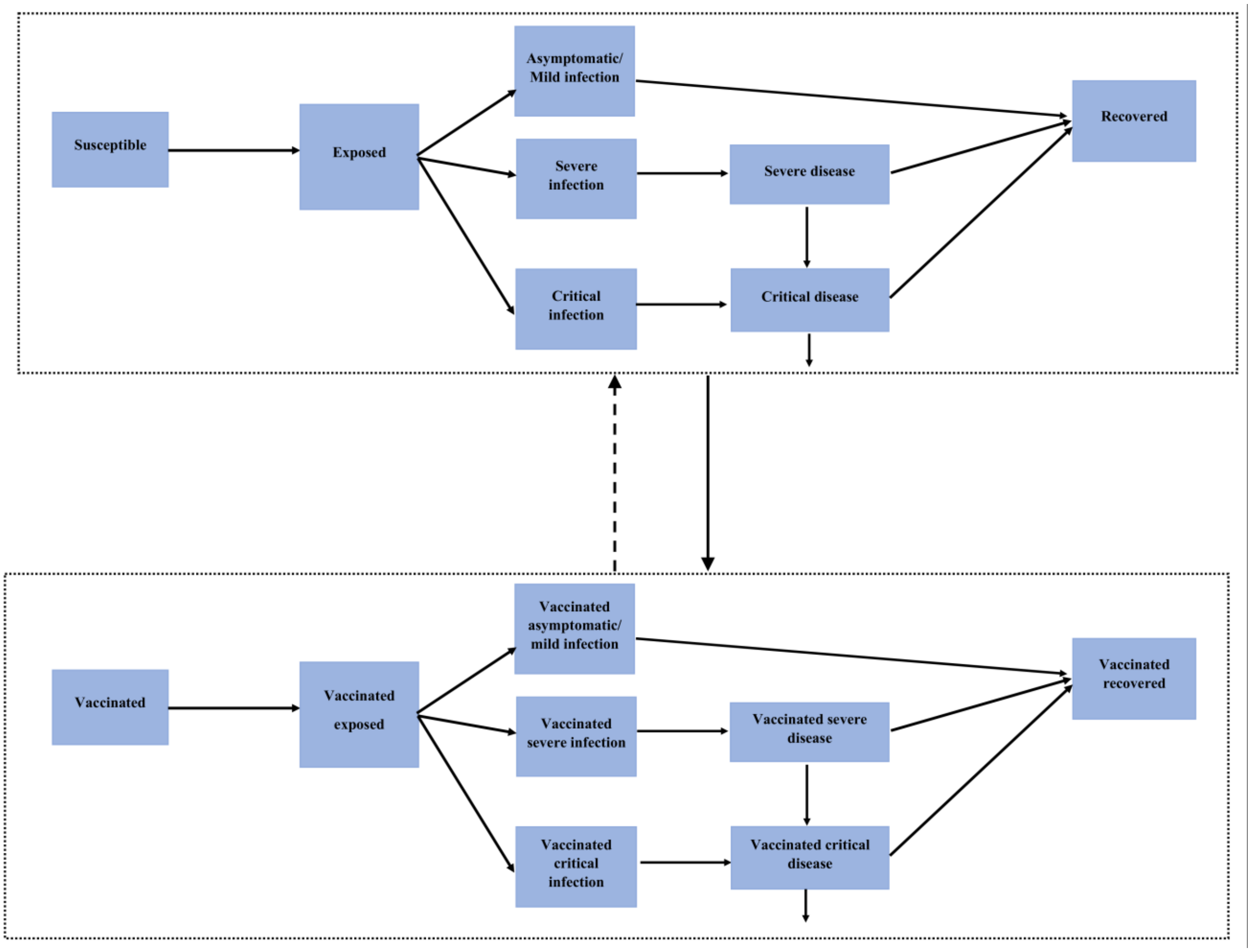

**Figure S2:** Bland-Altman plots showing agreement of the different $R_t^{Empirical}$ estimates generated through the four sensitivity analyses reported in Figure 2. Bland-Altman comparison of $R_t^{Empirical}$ calculated using A) the time series of all diagnosed cases versus only symptomatic cases, B) the time series of hospital admissions in acute-care beds versus symptomatic cases, C) the time series of hospital admissions in ICU-care beds versus symptomatic cases, D) different values for the generation time. The black line is the mean difference (bias) and the dashed red lines show the 95% limits of agreement. $R_t^{Empirical}$ was estimated using the Robert Koch Institute method [3].

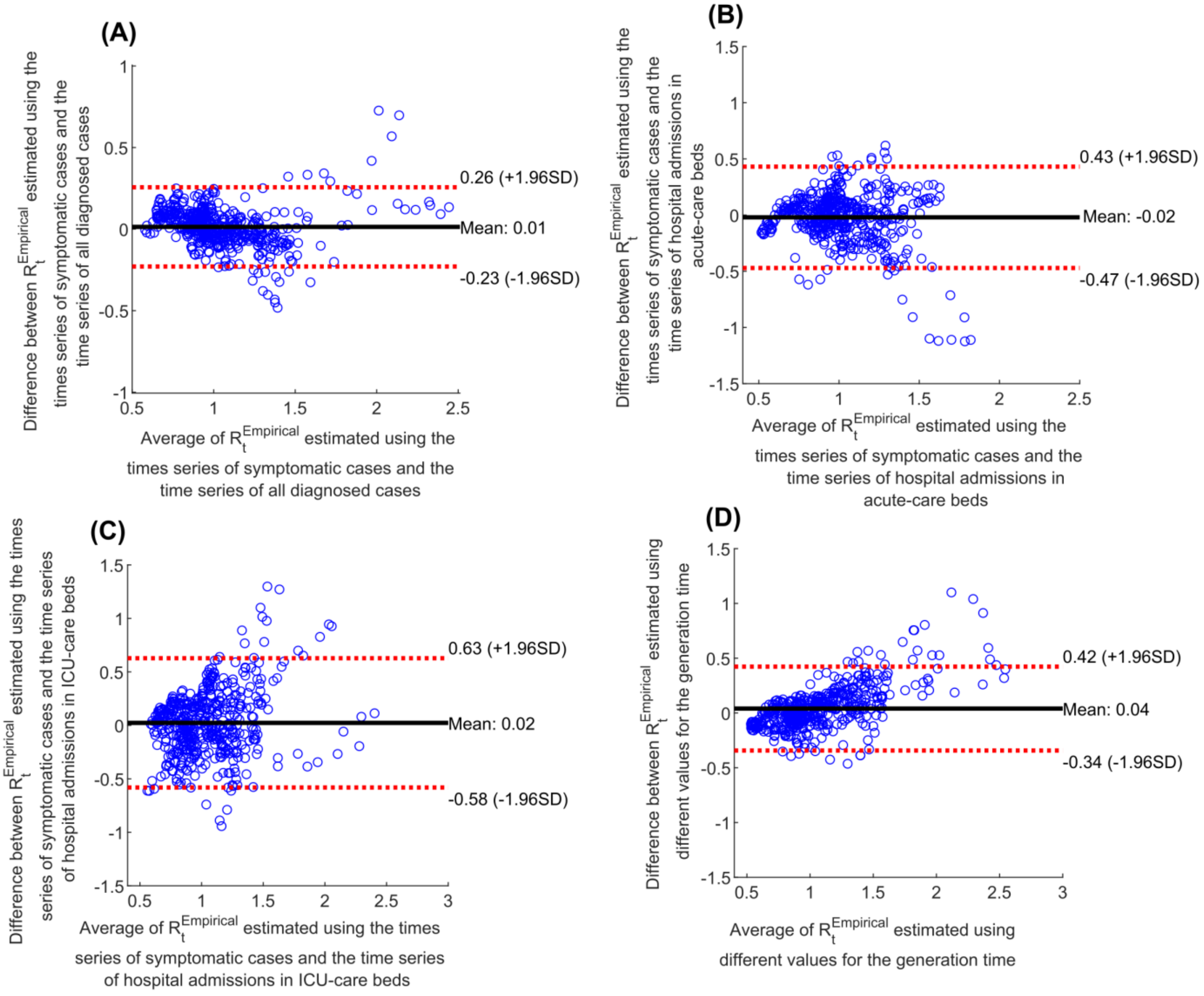

## References


1 Ayoub HH, Chemaitelly H, Makhoul M, Al Kanaani Z, Al Kuwari E, Butt AA, et al. Epidemiological impact of prioritising SARS-CoV-2 vaccination by antibody status: mathematical modelling analyses. BMJ Innovations. 2021:bmjinnov-2021-000677.

2 Ayoub HH, Chemaitelly H, Seedat S, Makhoul M, Al Kanaani Z, Al Khal A, et al. Mathematical modeling of the SARS-CoV-2 epidemic in Qatar and its impact on the national response to COVID-19. J Glob Health. 2021;11:05005.

3 Rober Koch Institut. Epidemiologisches Bulletin. 2020. Available at: https://www.rki.de/DE/Content/Infekt/EpidBull/Archiv/2020/Ausgaben/17_20.pdf?__blob=publicationFile. Accessed 28 May 2021.
